# Targeting the Wnt/β-Catenin Signaling Pathway as a Potential Therapeutic Strategy in Renal Tubulointerstitial Fibrosis

**DOI:** 10.3389/fphar.2021.719880

**Published:** 2021-08-16

**Authors:** Shan-Shan Li, Qian Sun, Meng-Ru Hua, Ping Suo, Jia-Rong Chen, Xiao-Yong Yu, Ying-Yong Zhao

**Affiliations:** ^1^ Department of Nephrology, Shaanxi Traditional Chinese Medicine Hospital, Xi’an, China; ^2^ The First School of Clinical Medicine, Shaanxi University of Traditional Chinese Medicine, Xianyang, China; ^3^ Faculty of Life Science and Medicine, Northwest University, Xi’an, China; ^4^ Department of Clinical Pharmacy, Affiliated Hospital of Chengdu University, Chengdu, China

**Keywords:** Wnt/β-catenin, chronic kidney disease, renal fibrosis, traditional Chinese medicine, natural product

## Abstract

The Wnt/β-catenin signaling pathway plays important roles in embryonic development and tissue homeostasis. Wnt signaling is induced, and *β*-catenin is activated, associated with the development and progression of renal fibrosis. Wnt/*β*-catenin controls the expression of various downstream mediators such as snail1, twist, matrix metalloproteinase-7, plasminogen activator inhibitor-1, transient receptor potential canonical 6, and renin-angiotensin system components in epithelial cells, fibroblast, and macrophages. In addition, Wnt/*β*-catenin is usually intertwined with other signaling pathways to promote renal interstitial fibrosis. Actually, given the crucial of Wnt/*β*-catenin signaling in renal fibrogenesis, blocking this signaling may benefit renal interstitial fibrosis. There are several antagonists of Wnt signaling that negatively control Wnt activation, and these include soluble Fzd-related proteins, the family of Dickkopf 1 proteins, Klotho and Wnt inhibitory factor-1. Furthermore, numerous emerging small-molecule *β*-catenin inhibitors cannot be ignored to prevent and treat renal fibrosis. Moreover, we reviewed the knowledge focusing on anti-fibrotic effects of natural products commonly used in kidney disease by inhibiting the Wnt/*β*-catenin signaling pathway. Therefore, in this review, we summarize recent advances in the regulation, downstream targets, role, and mechanisms of Wnt/*β*-catenin signaling in renal fibrosis pathogenesis. We also discuss the therapeutic potential of targeting this pathway to treat renal fibrosis; this may shed new insights into effective treatment strategies to prevent and treat renal fibrosis.

## Introduction

Chronic kidney disease (CKD) is an increasingly serious public health problem due to its high prevalence and mortality and greatly increases the risk of end-stage renal disease (ESRD), and cardiovascular disease (Webster et al., 2017). Renal fibrosis is the final pathological, dynamic, progressive, and irreversible process common to any ongoing CKD or maladaptive repair (Djudjaj and Boor, 2019). Renal fibrosis is the accumulation of scars in the parenchyma that is a pathological expansion of the normal wound healing process, characterized by inflammation, myofibroblast activation, migration, and matrix deposition and remodeling, leading to the replacement of functional parenchyma by fibrotic tissues (Humphreys, 2018; Distler et al., 2019). Renal interstitial fibrosis is mainly driven by various pro-fibrotic growth factors, forming a fibrotic micro-environment in the interstitial space (Chen et al., 2018c; Tang et al., 2019). In other words, the major pathological events of renal fibrosis include inflammatory cell infiltration, fibroblast activation and proliferation, and abnormal increase and excessive deposition of extracellular matrix (ECM) components, mainly composed of collagen, fibronectin, and proteoglycans (Liu et al., 2021). With the ECM continuous deposition, scar tissue replaces normal tissue, tubules, and peritubular capillaries are lost, resulting in disruption of tissue architecture and loss of renal function (Xing et al., 2021). Additionally, there is growing evidence that ECM-derived components could be used as danger-associated molecular patterns (DAMPs). As an important promoter of fibrogenesis, as long as the inflammatory stimulation persists, these DAMPs are generated and release signals during the phase cell activation and damage, ultimately promoting inflammation to fibrosis and kidney disease (Nastase et al., 2018).

The Wnt/*β*-catenin signaling pathway is an evolutionarily conserved developmental signaling pathway, playing an extremely important role in organogenesis, tissue homeostasis, and disease progression of multicellular organisms (Schunk et al., 2021). There are 19 identified encoding Wnt genes in the mammalian genome, all of which are cysteine-rich proteins (Langton et al., 2016). Wnt protein induces *β*-catenin-dependent signaling through Wnt receptor coiled Frizzled (FZD) and co-receptors low-density lipoprotein receptor-related protein-5/protein-6 (LRP5/6) (Janda et al., 2017). In addition, there are other receptors and co-receptors, including the tyrosine kinase receptors RYK, single transmembrane receptor tyrosine kinase, G-protein coupled receptor, etc., that trigger various downstream signaling pathways (Foulquier et al., 2018). Continuous accumulation of intracellular *β*-catenin signaling plays a crucial role in developing renal fibrosis, podocyte injury, proteinuria, persistent tissue damage during acute kidney injury, and cystic kidney diseases (Miao et al., 2019; Schunk et al., 2021). The latest research shows that given the crucial role of Wnt/β-catenin signaling in renal fibrogenesis, blocking this signaling may be beneficial to alleviate renal fibrosis (Xie et al., 2021; Yiu et al., 2021). Recent studies have shown that apigenin (API) could effectively relieve renal fibrosis via co-inhibiting uric acid (UA) reabsorption and the Wnt/*β*-catenin signaling pathway (Li et al., 2021). In addition, ischemia-reperfusion injury (IRI) could increase indoleamine-2,3-dioxygenase (IDO) expression to activate the Wnt/*β*-catenin pathway leading to renal fibrosis. Prostaglandin E2 (PGE2) could ameliorate kidney fibrosis via inhibiting IDO expression and reducing *β*-catenin resulting in lower expressions of α-smooth muscle actin (α-SMA), fibronectin (Pan et al., 2021). *In vivo* and *in vitro*, it has proved that the abnormally expressed cannabinoid receptor type 2 (CB2) is closely related to renal fibrosis via *β*-arrestin 1-induced β-catenin activation, and β-catenin could promote the activation and expression of CB2, and finally forms the vicious circle in the CB2/*β*-catenin pathway (Zhou et al., 2021). Therefore, it is of great significance to clarify the cellular and molecular mechanisms of the Wnt/*β*-catenin signaling pathway in tubulointerstitial fibrosis and provide a new treatment strategy for antifibrosis and delaying CKD progression. In this review, we summarize recent advances on the involvement of Wnt/*β*-catenin in the pathogenesis of tubulointerstitial fibrosis and the intervention effect of natural products targeting the Wnt/*β*-catenin signaling pathway.

## Wnt/*β*-Catenin Signaling Pathways

**FIGURE 1:**
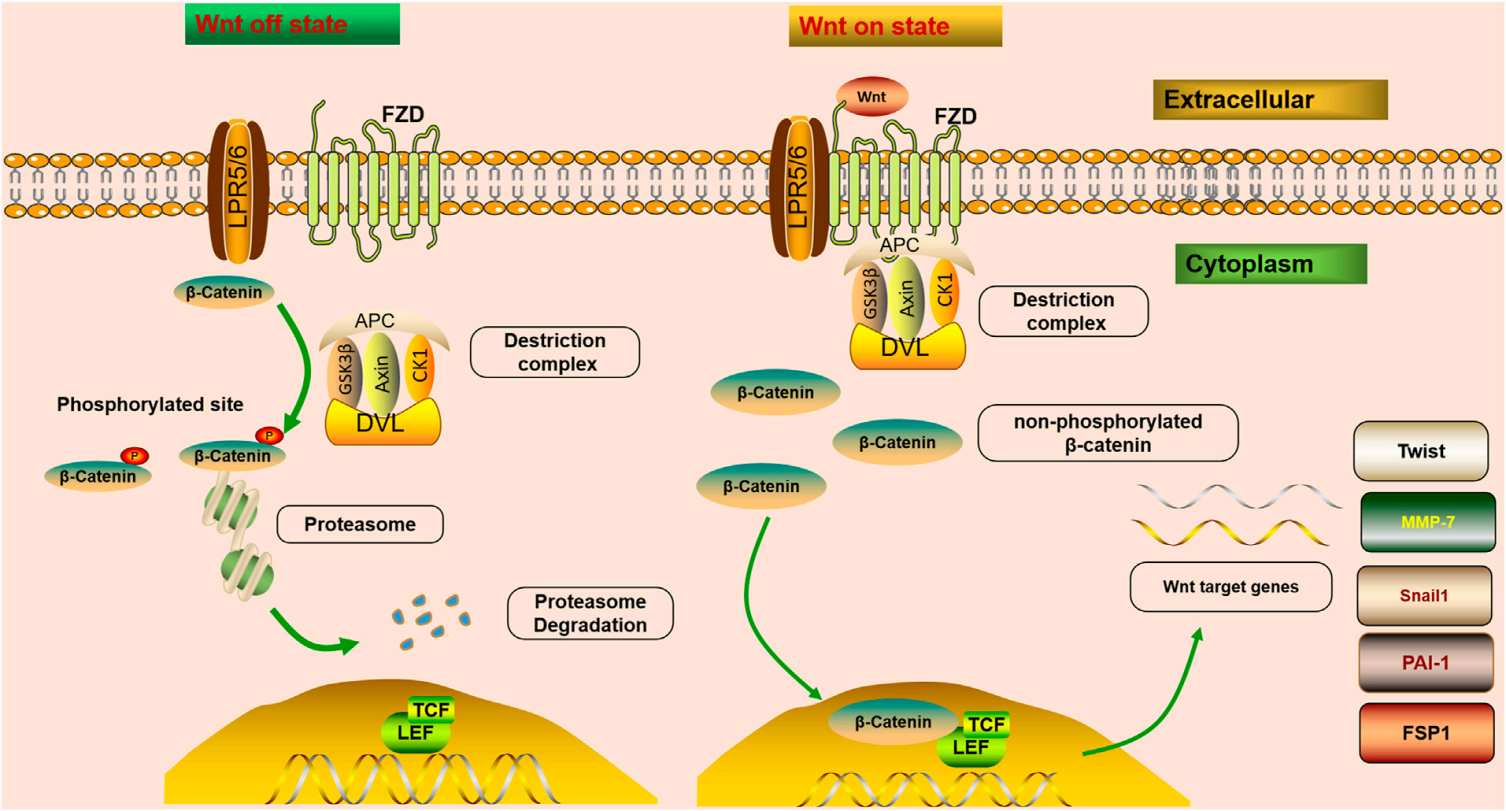
Wnt/*β*-catenin signaling is off or on state. In steady-state, *β*-catenin is inactivated by a “destruction complex,” and phosphorylation, ubiquitinylated, and degraded of *β*-catenin is mediated by the protein complex. Wnt on state; Wnt molecules transmit the intracellular signal in the intracellular matrix through interacting with FZD receptors and LRP5/6. After that, the interaction triggers the intracellular signal cascade and promotes the accumulation of non-phosphorylated *β*-catenin, which translocates into the nucleus, and cooperates with TCF/LEF to trigger the transcription of Wnt target genes. Once Wnt ligands bind to co-receptors on cytomembrane, the combination of FZD and DVL could provide a recruitment platform for the *β*-catenin destruction. Wnt/*β*-catenin controls the expression of various downstream mediators implicated in renal fibrosis, such as Snail1, MMP-7, PAI-1, Twist, and FSP1.

The mechanisms of Wnt signaling consist of two main branches: the canonical and non-canonical pathways (Schunk et al., 2021). The canonical pathway is also known as the Wnt/*β*-catenin pathway. Furthermore, two master non-canonical pathways are the Wnt/planar cell polarity pathway (Wnt/PCP pathway) and the Wnt/calcium pathway (Wnt/Ca^2+^ pathway) (Hu et al., 2020). According to downstream effects, all Wnt ligands are divided into two categories: one is canonical, Wnt ligands that induce *β*-catenin-dependent pathway, including Wnt1, 2, 3, 8a, 8b, 10a, and 10b, and the other is non-canonical, Wnt ligands that mediate *β*-catenin-independent pathway, including Wnt4, 5a, 5b, 6, 7a, 7b, and 11 (Acebron and Niehrs, 2016). In the canonical Wnt/*β*-catenin signaling pathway, Wnt molecules transmit the intracellular signal in the intracellular matrix through interacting with FZD receptors and the co-receptor LRP5/6 (Figure 1). FZD proteins have seven transmembrane receptors, with a cysteine-rich domain responsible for the binding of Wnt proteins (Schunk et al., 2021). Since the activation of LRP5/6 depends on the binding of the canonical Wnt ligands to the FZD receptors, they are considered to be the key co-receptors of the canonical Wnt signaling pathway (Huang et al., 2019). After that, the interaction triggers the intracellular signal cascade and promotes the accumulation of non-phosphorylated *β*-catenin, which translocates into the nucleus, and cooperates with the transcription factors T cell factor (TCF)/lymphatic enhancer-binding factor (LEF) to trigger the transcription of Wnt target genes (Tessa et al., 2020). *β*-Catenin is an important co-factor that binds multiple transcriptional molecules and mediates fibrogenic signaling pathways (Yang et al., 2021). In a steady-state, *β*-catenin is inactivated by a “destruction complex,” which includes the proteins glycogen synthase kinase 3*β* (GSK3*β*), adenomatous polyposis coli (APC), casein kinase 1 (CK1), and axin (Wang et al., 2020a). The protein complex mediates the phosphorylation, ubiquitinylation, and degradation of *β*-catenin, and through continuous ubiquitination and degradation, the cytoplasmic levels are kept at a low level (Schunk et al., 2021). However, once Wnt ligands bind to co-receptors on cytomembrane, the combination of FZD and Dishevelled (DVL) could provide a recruitment platform for the *β*-catenin destruction (Gammons et al., 2016). DVL protein is recruited, and the ‘destruction complex’ is disrupted, protecting *β*-catenin from inactivation and degradation, thus leading to the stabilization, accumulation, and nuclear translocation of *β*-catenin (Nusse and Clevers, 2017). An increasing number of studies have demonstrated that fibroblast signaling pathways all merge on *β*-catenin to promote the *β*-catenin/TCF complex and mediate fibrogenesis (Yang et al., 2021). *β*-Catenin integrates the inputs of transforming growth factor *β* (TGF-*β*)/Smad, integrin/ILK, the Wnt/*β*-catenin pathway and renin-angiotensin system (RAS), which are activated in fibrotic primary and allograft kidney diseases (Huang et al., 2019).

## Wnt/*β*-Catenin Signaling Pathway in Renal Fibrosis

The Wnt/*β*-catenin pathway is one of the crucial signaling pathways resulting in kidney disease. An increasing number of studies have demonstrated that the activation of the Wnt/*β*-catenin signaling pathway serves a key role in promoting renal fibrosis by controlling the expression of various downstream mediators implicated in renal fibrosis (Chen et al., 2017b; Feng et al., 2019a; Schunk et al., 2021) (Figure 1). The transient activation of many signaling pathways has a beneficial effect on repairing damaged tissues. However, their sustained activation promotes fibrosis (Edeling et al., 2016). It has been confirmed that severe ischemia/reperfusion injury leads to sustained and excessive activation of Wnt/*β*-catenin, accompanied by interstitial myofibroblast activation and ECM deposition, characteristics of renal fibrotic lesions development (Xiao et al., 2016). Therefore, sustained and exaggerated Wnt/*β*-catenin activation mediates fibroblast activation. Although transient Wnt/*β*-catenin activation promotes tissue regeneration and repair after kidney injury, sustained or uncontrolled Wnt/*β*-catenin signaling stimulates podocyte injury and proteinuria, ultimately leading to irreversible renal fibrosis (Schunk et al., 2021).

### Fibroblast

Fibroblasts are the main driving force for scar formation after kidney injury (Miao et al., 2021). The continuous activation of fibroblasts leads to the secretion of ECM components, such as collagen, proteoglycan, and fibronectin, leading to the development of renal fibrosis (Feng et al., 2019b). As an ECM glycoprotein, fibronectin serves a key role in wound healing and fibrosis by regulating the deposition of collagen and other ECM molecules. A study confirmed that upregulated Wnt/*β*-catenin signaling is related to the response of epithelial cells to wound, renal tubular cell damage, fibrous collagen, and immunoglobulin transcript expression (Venner et al., 2016). Thus, Wnt protein derived from renal tubules may play a critical role in fibroblast activation and renal fibrosis (Zhou et al., 2017). There is a complex regulatory network between renal tubular epithelial cells and fibroblasts, regulated by autocrine and paracrine Wnt/*β*-catenin signaling (Maarouf et al., 2016). The activated Wnt/*β*-catenin pathway can promote the fibroblast proliferation and differentiation of fibroblasts towards myofibroblasts; myofibroblasts are critical contributors to renal fibrosis. Their characteristics are secreting fibronectin and increasing the expression of α-SMA (Zhou et al., 2017). In a high glucose environment, activating the Wnt/*β*-catenin pathway could promote renal mesangial cell proliferation and fibronectin production. Interestingly, fibronectin is an important target gene of Wnt/*β*-catenin (Zhang et al., 2014). Therefore, inhibiting the activity of Wnt/β-catenin signaling in fibroblasts may help alleviate the progression of renal fibrosis (Duan et al., 2020).

### Macrophages

According to the activation mechanism and cell function, macrophages are divided into classically activated macrophages (M1) and alternatively activated macrophages (M2) (Wang et al., 2014a). M1, a pro-inflammatory phenotype, releases cytokines that inhibit the proliferation of surrounding cells and damage contiguous tissue, while M2, an anti-inflammatory phenotype, releases cytokines that promote the proliferation of contiguous cells and tissue repair (Wang et al., 2014a; Cosin-Roger et al., 2019). If macrophages fail to acquire a tissue-healing phenotype, dysregulated signals can be drivers of disease processes, such as sustained, exuberant inflammation and fibrosis (Smigiel and Parks, 2018). Thus, macrophages serve a key role in immune surveillance and in the maintenance of renal homeostasis. Macrophages recruited from the bone marrow can transition directly into myofibroblasts during renal injury. This process is defined as macrophage-to-myofibroblast transition, which may play a crucial role in the progression of chronic inflammation to pathogenic fibrosis (Tang et al., 2019). Sustained accumulation and activation of macrophages in kidney tissue could lead to the production of multiple pro-fibrotic cytokines and ultimately induce renal fibrosis. Additionally, as key inflammatory cells, macrophages could promote ECM synthesis and deposition, resulting in renal fibrosis by releasing inflammatory cytokines, TGF-*β,* and matrix-degrading enzyme inhibitors (Yang et al., 2019). Activation of Wnt/*β*-catenin signaling stimulates renal inflammation, comprising macrophages infiltration, pro-inflammatory cytokines release, and cell adhesion molecules expression in renal injury. Moreover, tubular cell-derived Wnt ligands mediate pro-inflammatory activation of renal macrophages during fibrosis (Wong et al., 2018). Previous studies have shown that the hyperactive of Wnt/*β*-catenin signaling could promote renal fibrosis by stimulating macrophage M2 polarization and promoting the proliferation and accumulation of macrophages. The continuous accumulation and activation of macrophages may lead to various fibrotic cytokines and ultimately lead to kidney fibrosis (Feng et al., 2018). Therefore, as an important source of Wnt protein in adult tissues, macrophages proliferate and accumulate in kidney tissues through the activation of the Wnt/*β*-catenin signal, considered a key factor in renal fibrosis (Cosin-Roger et al., 2019).

### Snail1

Snail family zinc finger 1 (Snail1) is a transcription factor expressed during embryonic renal development and is widely expressed in various kidney injury models, including unilateral ureteral obstruction (UUO), 5/6 nephrectomy, and hypoxia. It is involved in regulating fatty acid metabolism, cell cycle arrest, and inflammatory response, major biological processes responsible for renal fibrogenesis (Simon-Tillaux and Hertig, 2017). Snail1 is a key transcription factor driving epithelial-mesenchymal transition (EMT); the stabilized *β*-catenin enters the cell nucleus, forms a complex with TCF, and activates the transcription of Snail1 to drive EMT. Moreover, the up-regulation of TGF-*β*1 promotes Snail1-mediated EMT of renal tubular epithelial cells during renal fibrosis. Not only that, but also Snail1 is a critical transcription target of *β*-catenin that upregulates *β*-catenin transcriptional activity; as such, both *β*-catenin and Snail1 may be activated simultaneously to produce an additive or synergistic effect in promoting EMT (García de Herreros and Baulida, 2012). Thus, the Snail1-induced EMT process is a key mechanism that initiates the reaction cascade leading to fibrosis (Bai et al., 2016). Additionally, it has been found in a modern study that the Snail1/*β*-catenin signaling pathway may be involved in promoting renal fibrosis related to diabetes (Kim et al., 2017). Furthermore, it has been demonstrated that Snail1s could interact with *β*-catenin functionally, thereby increasing the expression of Wnt-dependent target genes (Stemmer et al., 2008).

### Matrix Metalloproteinase-7

MMP-7, also known as matrilysin, is a secreted zinc- and calcium-dependent endopeptidase, a transcriptional target of classic Wnt/*β*-catenin signaling, a pathological mediator, and therapeutic target of renal fibrosis (Wozniak et al., 2021). Under normal physiologic conditions, MMP-7 is almost not expressed in adult kidneys but upregulated in various renal diseases, including AKI and CKD (Wozniak et al., 2021). MMP-7 can degrade ECM components and cleave various substrates, such as E-cadherin, Fas ligand, and nephrin. Therefore, it plays a key role in regulating various biological processes, such as cell proliferation, apoptosis, EMT, and podocyte damage (Tan et al., 2019). Furthermore, MMP-7, via its proteolytic activity, mediates proteolytic degradation of E-cadherin, resulting in *β*-catenin liberation and activation, leading to renal fibrosis in a Wnt-independent fashion. It is worth noting that the release of *β*-catenin mediated by MMP-7 further induces the expression of MMP-7, eventually forming a vicious circle (Liu et al., 2020). In other words, on the one hand, activation of Wnt/*β*-catenin promotes the occurrence of fibrosis by upregulating pro-fibrotic mediators, including MMP-7, PAI-1. On the other hand, MMP-7, as the most powerful *β*-catenin downstream target, can activate the Wnt/*β*-catenin signaling pathway after renal injury (Tan et al., 2019). In summary, renal MMP-7 levels correlate with Wnt/*β*-catenin activity, and urinary MMP-7 may be a noninvasive biomarker of pro-fibrotic signaling in the kidney. However, in several AKI animal models (IRI, cisplatin administration, and folic-acid induced AKI), MMP7 exerts protective effects on the kidney as an adaptive response. Therefore, the role of MMP-7 as a therapeutic target for kidney disease needs further study.

### Plasminogen Activator Inhibitor-1

PAI-1, as a member of the serine protease inhibitor family, interferes with ECM and fibrin degradation, mediated by urokinase-type plasminogen activator (uPA) and tissue-type plasminogen activator (tPA) to suppress fibrinolysis and contribute to interstitial fibrosis in the kidney injury (Flevaris and Vaughan, 2017). Increasing evidence shows the role of PAI1 in renal fibrosis (Zhou et al., 2015a). PAI-1 promotes fibrosis by participating in various cellular processes, such as inflammation, cell adhesion, and migration. Conversely, some specific factors that promote fibrosis, such as oxidative stress and hypoxia, could also affect the expression of the PAI-1 gene (Rabieian et al., 2018). And depleting PAI-1 can alleviate interstitial fibrosis by decreasing fibroblast activation and proliferation in the renal interstitium (Yao et al., 2019). Therefore, PAI-1, a key molecule in renal fibrosis progression, has increased expression in various kidney disease models (Hamasaki et al., 2013). In addition, a recent study indicated that PAI-1 could promote cell migration through LRP1-dependent *β*-catenin activation (Kozlova et al., 2015). Since the promoter region of PAI-1 contains a TCF/LEF binding site, PAI-1 is an important target gene of *β*-catenin signaling in renal injury (Malik et al., 2020).

### Components of the Renin-Angiotensin System

The RAS serves a vital role in maintaining renal hemodynamics and the occurrence of hypertension and kidney disease (Navar, 2014). Unanimously, renal tissue RAS has various pathophysiological functions in regulating blood pressure, growth of kidney cells, and glomerular sclerosis, leading to renal fibrosis development (Urushihara et al., 2012). The (pro)renin receptor ((P)RR), consisting of 350 amino acids, has been considered as a single-transmembrane protein encoded by ATP6AP2, an X chromosome-located gene, and the transmembrane receptors enhance the tissue RAS by binding to their ligands renin and/or prorenin; therefore, it is initially considered to be an important part of the RAS and is ubiquitously expressed in the human body (Ramkumar and Kohan, 2016; Ichihara and Yatabe, 2019). The receptor plays crucial roles in various pathways, involved in extensive physiological and pathological processes, such as the cell cycle, autophagy, acid-base balance, energy metabolism, T cell homeostasis, blood pressure regulation, cardiac remodeling, and maintaining podocyte structure (Ichihara and Yatabe, 2019; Wang et al., 2020a). The Wnt-RAS signaling serves a vital role in the development and progression of CKD (Zhou et al., 2020). There are putative TCF/LEF binding sites in the RAS promoter region through bioinformatics analyses, and *β*-catenin could trigger LEF-1 to bind to these sites in renal tubular cells (Zhou et al., 2015b). In addition, (P)RR is necessary for signal transduction through FZD-LRP5/6 (Li et al., 2017). Moreover, accumulating evidence has demonstrated that (P)RR is a downstream target and a crucial element in Wnt signal transmission, promoting kidney damage and fibrosis through amplifying Wnt/*β*-catenin signaling transduction. In addition, Wnt/*β*-catenin, as the main upstream regulator, controls the expression of multiple RAS genes. In other words, the overactivity of *β*-catenin or different Wnt ligands leads to the expression of all RAS genes (Zhou and Liu, 2015; Zhao et al., 2018). Hence, targeting Wnt/*β*-catenin would concurrently inhibit all RAS genes, accordingly suppressing inflammation and alleviating renal fibrosis (Zhou and Liu, 2016). Studies have demonstrated that the fibrogenic action of Wnt/*β*-catenin is dependent on RAS activation, and Wnt/*β*-catenin regulates multiple RAS genes. At the same time, RAS can induce the expression of multiple Wnt genes *in vivo* and *in vitro*; as such, the Wnt/*β*-catenin-RAS axis can be known as a vicious circle in aggravating the renal injury (Xiao et al., 2019).

### Transient Receptor Potential Canonical 6

TRPC6 has been implicated in the pathogenesis of kidney diseases, including focal segmental glomerulosclerosis (FSGS), diabetic nephropathy (DN), immune-mediated kidney disease, and renal fibrosis. As a result, TRPC6 has become a critical target of therapeutic agents to prevent and treat various kidney diseases (Hall et al., 2019). TRPC6 is another transcriptional target of the Wnt/*β*-catenin signaling cascade, and the Wnt/*β*-catenin signaling pathway may potentially be active in the pathogenesis of TRPC6-mediated diabetic podocyte injury (Zhang et al., 2013; Kim and Dryer, 2021). TRPC6 knockout shows protection on UUO-triggered kidney tubulointerstitial injury, interstitial fibrosis, and α-SMA expression (Gu et al., 2020). Mutations and over-activation in TRPC6 channel activity play an important role in podocyte damage in DN(Staruschenko et al., 2019; Wang et al., 2020c), However its role in renal fibrosis and the interaction with the Wnt signaling pathway in renal fibrosis still need further study.

In summary, the Wnt/β-catenin signaling pathway could activate renal fibrosis-related cytokines and up-regulate the expression of downstream target genes, eventually inhibit the main pathological process in the fibrosis process, and improve renal fibrosis. Therefore, we can further explore the downstream targets of the Wnt/β-catenin signaling pathway on this basis to further improve the mechanism of the Wnt/β-catenin signaling pathway in renal fibrosis.

## The Crosstalk Between Wnt/*β*-Catenin and Other Signaling Pathways on Renal Interstitial Fibrosis

Various pathways intersect and regulate each other to induce appropriate responses to a series of complex stimuli. Therefore, the synergy between other pathological signaling pathways and Wnt may play an important role in promoting renal fibrosis. RNA sequencing showed that deleting TGF-*β* receptors in proximal renal tubular cells regulated many growth factor pathways, but Wnt/*β*-catenin signaling is the most affected pathway due to the activity of *β*-catenin that is impaired *in vivo* and *in vitro* (Nlandu-Khodo et al., 2017). The participation of the Wnt signaling pathway enhances the pro-fibrotic effect of the TGF-*β* signaling pathway (Yang et al., 2020). In hypoxic pathological damage of organs, overactivation hypoxia-inducible factor-1*α* (HIF-1*α*) activates the Wnt/*β*-catenin signaling pathway, thereby aggravating renal interstitial fibrosis development (Qi et al., 2017). Although the Hedgehog (HH) signaling pathway is considered upstream of the Wnt/*β*-catenin signaling pathway, there is overlap between the two phenotypic results, suggesting a synergistic effect. Wnt and Notch interact mostly synergistically in the stem cell and epithelial cell compartment to trigger fibrosis development via suppressing epithelial differentiation; Notch, as a negative regulator of the Wnt/*β*-catenin signaling pathway, promotes *β*-catenin degradation by establishing a complex with *β*-catenin (Edeling et al., 2016; Chatterjee and Sil, 2019). However, the mechanism of the interaction between the signaling pathways is still unclear, and the studies on the mechanism of their synergy with the Wnt/*β*-catenin signaling pathway should be further explored.

## Targeting Wnt/*β*-Catenin Signaling as a Therapeutic Potential for Renal Fibrosis

Accumulating evidence has demonstrated that inhibiting the Wnt signaling pathway could alleviate renal interstitial fibrosis by attenuating apoptosis and expression of fibrosis-associated markers in renal cells (Ren et al., 2019; Cai et al., 2020; Huang et al., 2020). Endogenous Wnt inhibitors can negatively regulate the Wnt signaling pathway by binding to Wnt ligands competitively with Wnt receptors or co-receptors, such as Dickkopf1 (DKK1), secreted frizzled protein 1 (Sfrp1), Wnt inhibitor 1 (Wif-1), Klotho (Kawazoe et al., 2021). In addition, exogenous Wnt signaling inhibitors accompanied by natural products cannot be ignored to prevent and treat renal fibrosis. Collectively, the Wnt/*β*-catenin signaling pathway may serve as a potential treatment strategy for renal fibrotic disorders.

### Endogenous Wnt Inhibitors

#### Secreted Frizzled-Related Protein1

Humans have five secreted frizzled-related proteins (Sfrp1-5) with cysteine-rich domains (CRD), and these Sfrps have a strong homology with FZD receptors, therefore compete with FZD receptors for Wnt binding. In other words, Sfrps act as a Wnt inhibitor. The Sfrp family consists of secreted glycoproteins that can competitively bind to Wnt, inhibiting the canonical and non-canonical Wnt signaling pathways (Cruciat and Niehrs, 2013). Studies have confirmed that Sfrp1 regulates cell proliferation and differentiation by regulating Wnt/*β*-catenin signaling, showing low expression in various tumor tissues (Qiao et al., 2017). Thus, Sfrp1 is a Wnt antagonist that acts as a negative regulator of Wnt/*β*-catenin signaling and serves a key role in fibrotic diseases. In the mouse model of UUO, knockout of Sfrp1 significantly increases the expression of *α*-SMA and the protein level of vimentin; meanwhile, it decreases the protein level of E-cadherin, which enhances the epithelial to mesenchymal transition (Matsuyama et al., 2014). Additionally, down-regulation of Sfrp1 activates the Wnt/*β*-catenin signaling pathway, increased ECM deposition, eventually lead to renal fibrosis. Therefore, Sfrp1 acts as a negative regulator of the Wnt signaling pathway and suppresses renal fibrosis via inhibiting the Wnt/*β*-catenin signaling pathway.

#### Klotho

Klotho, an anti-aging protein, reduces renal fibrosis after AKI. Klotho serves a key role in regulating various cellular processes by interacting with multiple signaling molecules, including oxidative stress, fibrosis, inflammation, autophagy, and apoptosis (Hu et al., 2013). Therefore, Klotho is a critical gene, controlling aging and kidney homeostasis, and an ideal intervention target for various kidney diseases and even extrarenal complications (Xia and Cao, 2021). Furthermore, Klotho plays an important role in anti-fibrotic activities by inhibiting oxidative stress and excessive inflammation. Hence, Klotho deficiency enhances renal fibrosis (Lindberg et al., 2014). It has been shown that the extracellular domain of Klotho inhibits Wnt signaling via binding to multiple Wnt ligands (Muñoz-Castañeda et al., 2020). Thus, Klotho is a critical negative regulator of canonical Wnt signaling and suppresses renal fibrosis in the obstructed kidney model by simultaneously suppressing multiple growth factor signaling pathways such as fibroblast growth factor-2 (FGF-2), Wnt, and TGF-*β*1 (Guan et al., 2014). A study has found that Klotho represses the Wnt/*β*-catenin pathway in renal tubular epithelial cells (TECs) to exert a stronger anti-fibrotic effect (Zhang et al., 2018a). Wnt/*β*-catenin activation is considered to be the key factor resulting in Klotho downregulation (Muñoz-Castañeda et al., 2017). In addition, the upregulation of Klotho prevents Wnt activation, thereby inhibiting the deposition of ECM and reducing the transcription of cytokines, ultimately improving renal fibrosis (Zhou L. et al., 2013). Therefore, Klotho is termed an antagonist of endogenous Wnt/*β*-catenin activity, and increasing Klotho levels could be a strategy to reduce the morbidity and mortality of kidney-related diseases (Muñoz-Castañeda et al., 2020).

#### Dickkopf 1

DKK1 is an important member of the DKK family (DKK1, DKK2, DKK3, DKK4) and is widely expressed in many fields. It has been considered a secreted protein that can suppress the Wnt signaling transduction pathway (Huang et al., 2018). DKK1 may play a crucial role in the fibrotic process in several organs such as the liver, lungs, and kidneys (Klavdianou et al., 2017). Gene therapy using DKK1 significantly suppresses fibroblast-specific protein 1(fsp1), a marker for fibroblasts and myofibroblasts, type I collagen, and fibronectin mRNA in the model of obstructive nephropathy, thereby repressing the activation of myofibroblast and improving renal fibrosis (He et al., 2009). *In vivo*, DKK1 effectively inhibited inflammation and fibrosis associated with ureteral obstruction (Johnson et al., 2017). DKK1 can inhibit the canonical Wnt signaling pathway through binding to LRP5/6, as well as interrupting the formation of the LRP and Wnt protein complex. (Hou et al., 2021), Therefore, DKK1 is termed an inhibitor of canonical Wnt/*β*-catenin signaling (Lipphardt et al., 2019). As a Wnt antagonist, DKK1 blocks Wnt-mediated fibrosis and also down-regulates its expression under fibrotic conditions. Therefore, it is termed a comprehensive regulator of the Wnt signaling pathway and has been proven to participate in renal fibrosis, glucose metabolism, and inflammation (Huang et al., 2018). Overall, there is no doubt that the outlook for DKK1 target therapy is promising. DKK1, DKK2, and DKK4 act as Wnt antagonists by directly binding to LRP5/6, thereby inhibiting Wnt/*β*-catenin-dependent signaling (Joiner et al., 2013). However, in UUO rat models and adenine-induced nephropathy, DKK3 is a major driver of renal fibrosis (Federico et al., 2016), and the exact role of DKK3 on Wnt/*β*-catenin signaling remains poorly unclear.

#### Wnt Inhibitor Factor 1

Wif-1 is an antagonist of the Wnt signaling pathway, inhibiting the Wnt signaling pathway by binding to the Wnt ligand. Hypermethylation of the Wif-1 promoter leads to down-regulation of Wif-1 expression, which activates the Wnt signaling pathway, further promotes cell proliferation, and induces cell apoptosis (Lin et al., 2017). On the contrary, the recovered Wif-1 expression level inhibits the Wnt signaling pathway. In fibroblasts from Systemic sclerosis (SSc) patients, an autoimmune disease characterized by extensive visceral organ and skin fibrosis, expression of Wif-1 is decreased. And knockdown of Wif-1 in normal fibroblasts induces Wnt signaling and collagen production (Svegliati et al., 2014). In the prevention and treatment of various diseases, Wif-1 plays an important role in inhibiting cell proliferation and migration by inhibiting the Wnt/*β*-catenin signaling pathway, but its role in renal fibrosis needs further study.

### Exogenous Wnt Inhibitors

**FIGURE 2:**
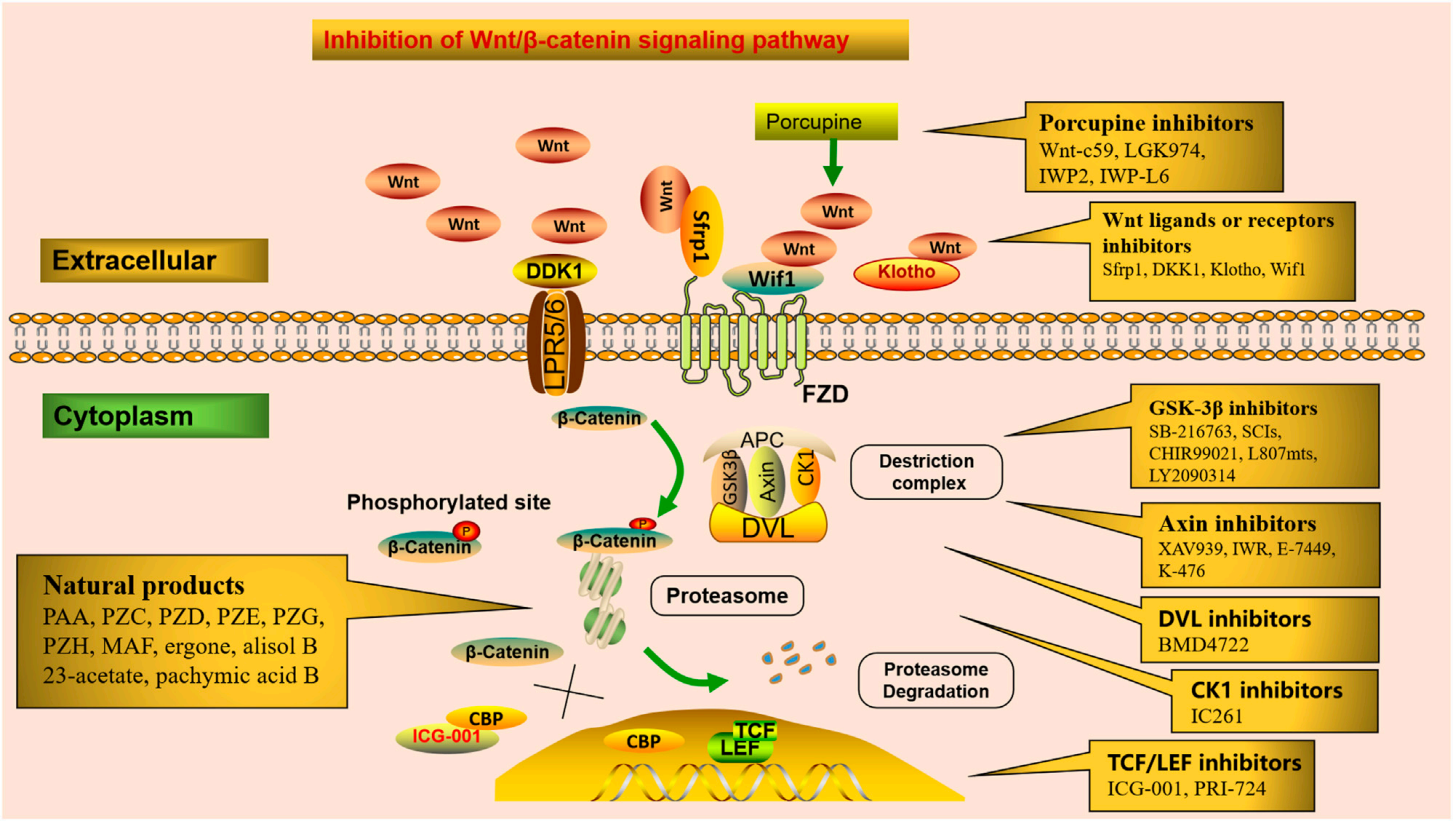
Potential therapeutic targets in the Wnt/*β*-catenin signaling. Numerous small molecules inhibit Wnt/*β*-catenin signaling at different steps of the pathway. Porcupine inhibitors prevent Wnt ligands secretion. Receptor or co-receptor inhibitors prevent the receptor actions of Wnt ligands. Inhibitors of GSK3*β*, axin, DVL, and CK1 interfere with Wnt/*β*-catenin downstream signaling. Inhibitors of TCF/LEF suppress Wnt/*β*-catenin-dependent gene transcription. Several natural products alleviate renal fibrosis by regulating the Wnt/*β*-catenin signaling pathway.

The Wnt/*β*-catenin signaling pathway can be therapeutically targeted at several steps (Figure 2). Firstly, blocking the production of all active Wnts. Porcupine (PORCN) is a membrane-bound O-acyltransferase required for Wnt palmitoylation, secretion, and biologic activity, and PORCN inhibitors prevent the release of Wnt ligands, such as Wnt-c59, LGK974(Wnt-974), IWP2, and IWP-L6 (Moon et al., 2017). Secondly, inhibitors of GSK3*β*, axin, DVL, and CK1 interfere with Wnt/*β*-catenin downstream signaling. Numerous studies have shown that GSK-3*β* activation plays a wide spectrum of important roles in tissue fibrosis (Zhuang et al., 2018; Zeng et al., 2019). SB-216763, a GSK-3*β* inhibitor, protects against Aldo-induced renal injury by activating autophagy and might be a therapeutic option for renal fibrosis (Zhang et al., 2018b). Substrate competitive inhibitors (SCIs), a novel small molecule GSK-3 inhibitor, is considered to be highly selective and more suitable for clinical practice (Rippin et al., 2020). In addition, CHIR99021 (Ng-Blichfeldt et al., 2019), L807mts, (Licht-Murava et al., 2016) and LY2090314 (Tu et al., 2017) have been produced as emerging small molecule inhibitors of GSK-3*β*. Tankyrase (Tnks) is a transferase that targets axin for proteasome degradation, and loss of Tnks activity leads to accelerated *β*-catenin destruction. XAV939 and inhibitors of Wnt response (IWR) act as Tankyrase inhibitors, which ultimately promote *β*-catenin degradation and inhibit Wnt/*β*-catenin signaling transcription (Kulak et al., 2015). In addition, E-7449 (Plummer et al., 2020) and K-476 (Kinosada et al., 2021), as new inhibitors of Tnks, have been widely used in anti-tumor therapy, and their anti-fibrotic mechanism needs to be further studied. BMD4722 specifically inhibits DVL by inhibiting the protein-protein interaction approach (Ma et al., 2018). IC261 is discovered as an ATP-competitive inhibitor of CK1 (Xian et al., 2021). Finally, inhibitors of TCF/LEF prevent Wnt/*β*-catenin-dependent gene transcription. CREB-binding protein (CBP) acts as a co-activator of multiple transcription factors for Wnt signal transduction formed with TCF. ICG-001, a small-molecule *β*-catenin inhibitor, inhibits the canonical Wnt/*β*-catenin signaling pathway by binding to CBP and blocking the interaction between *β*-catenin and CBP (Akcora et al., 2018). It inhibits renal tubular EMT by suppressing the transcription of a series of *β*-catenin-driven genes, such as Snail1, PAI-1, collagen I, fibronectin, and RAS, thereby ameliorates interstitial myofibroblast activation, represses matrix deposition, relieves proteinuria, ameliorates kidney inflammation, and alleviates fibrosis and exerts renal protection (Xiao et al., 2019). In a UUO murine model, ICG-001 could reduce the macrophage-to-myofibroblast transition of bone marrow-derived macrophages in renal fibrosis by inhibiting the *β*-catenin/TCF interaction (Yang et al., 2019). Therefore, ICG-001 not only prevents but reverses established fibrosis (Henderson et al., 2010). Moreover, PRI-724, as a selective inhibitor of CBP/*β*-catenin interaction, specifically destroys the interaction between *β*-catenin and CBP and has an encouraging effect in anti-liver fibrosis (Nishikawa et al., 2018).

### Specific Inhibition of Wnt/*β*-Catenin Signaling by Natural Products

Modern clinical pharmacological studies have confirmed that Chinese herbal medicines (CHMs) have a wide range of biological activities and play a broad and important role in regulating immune function by exerting their anti-cancer, anti-inflammatory, and anti-fibrosis effects (Zhao, 2013; Chen et al., 2016; Chen et al., 2018a; Wu et al., 2021). Clinical trials and experimental studies have shown that CHMs have great advantages in reducing proteinuria and improving renal function, by focusing on the anti-inflammatory, anti-oxidative, anti-apoptotic and anti-fibrotic effects (Chen et al., 2019a; Miao et al., 2020; Wang et al., 2020b; Wang et al., 2021a; Wang et al., 2021b). In addition, CHMs play a vital role in alleviating renal fibrosis by regulating the Wnt/*β*-catenin signaling pathway (Liu et al., 2019; Dai et al., 2020).

“Concept of holism” and “treatment based on syndrome differentiation” are the basic principles of traditional Chinese medicine (TCM) throughout the treatment of diseases. A study confirmed that *Qishen Yiqi dripping pill (QYDP)* reduces the renal Wnt1, *β*-catenin, TGF-*β*1, and Smad2 gene expression and downregulates collagen I, *α*-SMA, and fibronectin expression significantly in diabetic rats. The study results showed that *QYDP* ameliorates kidney function and renal fibrosis in diabetic rats by repressing the Wnt/*β*-catenin and TGF-*β*/Smad2 signaling pathways (Zhang et al., 2020). *Qingshen Buyang Formula* significantly reduces the expression of collagen I and fibronectin, the main components of ECM. Furthermore, by inhibiting EMT and Wnt/*β*-catenin signaling pathway, it improves renal injury and relieves renal fibrosis (Zhang et al., 2019). *Zhen-Wu-tang* alleviates adenine-induced chronic renal failure (CRF) by regulating the canonical Wnt4/*β*-catenin signaling, associated with improvement of renal fibrosis because it suppresses the overexpression of collagen IV and fibronectin, two key components of fibrosis (La et al., 2018). *Huang Gan Formula* is a new prescription developed and simplified based on *uremia clearing granule* and the theoretical basis of TCM. *HGF* inhibits the Wnt/*β*-catenin signaling pathway, significantly reducing glomerulosclerosis and tubular interstitial fibrosis and improving residual renal function (Mo et al., 2015).

Several compounds isolated from natural products promote urination and eliminate edema, which greatly benefits renal disease and fibrosis (Zhao et al., 2009; Zhao et al., 2012a; Zhao L. et al., 2013; Zhao Y.-Y. et al., 2013; Tian et al., 2014; Zhao et al., 2014). As an edible mushroom, *Poria Cocos* is widely used for diuretic, anti-inflammatory, antioxidant, lipid-lowering, and anti-fibrotic effects (Zhao et al., 2012b; Feng et al., 2013; Wang et al., 2013b; Miao et al., 2016; Chen et al., 2019b; Feng et al., 2019c). Poricoic acid A (PAA), as the main triterpenoid compound of *Poria Cocos*, exhibits renoprotective effects, (Chen et al., 2019c; Feng et al., 2019a; Chen et al., 2020a) and PAA showed anti-fibrotic effects via regulating the Wnt/*β*-catenin pathway (Chen et al., 2019d). In the HK-2 cell and UUO model, *poricoic acid ZC (PZC)*, *poricoic acid ZD (PZD),* and *poricoic acid ZE (PZE)* could alleviate renal fibrosis by effectively blocking RAS by simultaneously targeting multiple RAS components, correlated with activation of Wnt/*β*-catenin pathways (Wang et al., 2018a). Additionally, *poricoic acid ZG (PZG)* and *poricoic acid ZH (PZH)* significantly suppress the activation of Wnt/*β*-catenin signaling and relieve renal fibrosis (Wang et al., 2018b). 25-O-methylalisol F is a new tetracyclic triterpenoid compound isolated from the *Alismatis rhizome* that exhibits renoprotective effects (Chen et al., 2014; Feng et al., 2014; Tian et al., 2014; Dou et al., 2018), and is a novel RAS inhibitor by simultaneously targeting multiple RAS components (Chen et al., 2018b). Ergone, alisol B 23-acetate, and pachymic acid B inhibit ECM accumulation, suppress oxidative stress and inflammation, and regulates the Wnt/*β*-catenin signaling pathway (Zhao et al., 2011; Chen et al., 2017a; Chen et al., 2019e; Chen et al., 2020b).

*Curcumin* alleviated ECM accumulation in diabetic nephropathy by down-regulating Wnt/*β*-catenin signaling and rescued diabetic renal injury (Ho et al., 2016). *Salvia miltiorrhiza* extracts relieve renal injury by suppressing the relative expression levels of wnt4, *β*-catenin, and TGF-*β* in renal tissue (Xiang et al., 2019). *Salidroside* protects against T1DM-induced kidney injury and renal fibrosis by ameliorating TGF-*β*1 and the Wnt1/3a/*β*-catenin signaling pathway (Shati and Alfaifi, 2020). It has been demonstrated that *Tripterygium wilfordii* treatment inhibits the upregulation of Wnt1 and *β*-catenin expression in hyperglycemia-induced kidney tissue and attenuates the renal injury in rats caused by diabetes (Chang et al., 2018). *Triptonide* can effectively inhibit canonical Wnt/*β*-catenin signaling by targeting the downstream C-terminal transcription domain of *β*-catenin or a nuclear component associated with *β*-catenin (Chinison et al., 2016). *Astragaloside IV* has been shown to have possible inhibitory effects on renal interstitial fibrosis by effectively inhibiting the upregulation of proteins in the Wnt/*β*-catenin signaling pathway in UUO model rats (Wang et al., 2014b). Quercetin inhibits *β*-catenin signaling transduction, thereby inhibiting the activation of fibroblasts and renal fibrosis (Ren et al., 2016).

In short, based on the findings described above, the Wnt/*β*-catenin signaling pathway can be therapeutically targeted at several steps. Lots of emerging small molecule inhibitors that target Wnt and/or *β*-catenin are under development (Table 1). Additionally, the natural products, active ingredients, crude extracts, and traditional Chinese medicine formulas play an important role in anti-kidney fibrosis by inhibiting the Wnt/*β*-catenin signaling pathway. However, the specific molecular mechanisms of numerous inhibitors still need in-depth study, and more inhibitors need to be further developed.

**TABLE 1:** Summary of small molecular inhibitors of Wnt/*β*-catenin signaling.

Inhibitors	Targets	Effect	Reference(s)
Sfrp1	FZD	Ameliorates renal fibrosis	Henderson et al. (2020)
Klotho	Wnt ligands	Ameliorates renal fibrosis	Zhang et al. (2018a)
DDK1	LRP5/6	Ameliorates renal fibrosis	Lipphardt et al. (2019)
Wif1	Wnt ligands	Ameliorates fibrosis	Lin et al. (2017)
Wnt-c59	PORCN	Prevents Wnt ligands secretion	Proffitt et al. (2013)
LGK974	PORCN	Prevents Wnt ligands secretion	Moon et al. (2017)
IWP2	PORCN	Prevents Wnt ligands secretion	Wang et al. (2013a)
IWP-L6	PORCN	Prevents Wnt ligands secretion	Wang et al. (2013a)
SB-216763	GSK-3*β*	Inhibits Wnt signaling	Zhang et al. (2018b)
SCIs	GSK-3*β*	Inhibits Wnt signaling	Rippin et al. (2020)
CHIR99021	GSK-3*β*	Inhibits Wnt signaling	Ng-Blichfeldt et al. (2019)
L807mts	GSK-3*β*	Inhibits Wnt signaling	Licht-Murava et al. (2016)
LY2090314	GSK-3*β*	Inhibits Wnt signaling	Tu et al. (2017)
XAV939	Tnks and axin	Inhibits Wnt signaling	Kulak et al. (2015)
IWR	Tnks and axin	Inhibits Wnt signaling	Kulak et al. (2015)
E-7449	Tnks and axin	Inhibits Wnt signaling	Plummer et al. (2020)
K-476	Tnks and axin	Inhibits Wnt signaling	Kinosada et al. (2021)
BMD4722	DVL	Inhibits Wnt signaling	Ma et al. (2018)
IC261	CK1	Inhibits Wnt signaling	Xian et al. (2021)
ICG-001	CBP	Inhibits Wnt signaling	Akcora et al. (2018)
PRI-724	CBP	Inhibits Wnt signaling	Nishikawa et al. (2018)
PAA	Wnt/*β*-catenin signaling	Ameliorates fibrosis	Chen et al. (2019a)
PZC	Wnt/*β*-catenin signaling	Ameliorates fibrosis	Wang et al. (2018a)
PZD	Wnt/*β*-catenin signaling	Ameliorates fibrosis	Wang et al. (2018b)
PZE	Wnt/*β*-catenin signaling	Ameliorates fibrosis	Wang et al. (2018a)
PZG	Wnt/*β*-catenin signaling	Ameliorates fibrosis	Wang et al. (2018a)
PZH	Wnt/*β*-catenin signaling	Ameliorates fibrosis	Wang et al. (2018b)
MAF	Wnt/*β*-catenin signaling	Inhibits RSA	Chen et al. (2018a)
Ergone	Wnt/*β*-catenin signaling	Repress ECM accumulation	Chen et al. (2017a)
Alisol B 23-acetate	Wnt/*β*-catenin signaling	Repress ECM accumulation	Chen et al. (2017b)
Pachymic acid B	Wnt/*β*-catenin signaling	Repress ECM accumulation	Chen et al. (2017a)
Curcumin	Wnt/*β*-catenin signaling	Repress ECM accumulation	Ho et al. (2016)
Salvia	Wnt/*β*-catenin signaling	Relieve renal injury	Xiang et al. (2019)
Salidroside	Wnt/*β*-catenin signaling	Ameliorates fibrosis	Shati and Alfaifi (2020)
TW	Wnt/*β*-catenin signaling	Relieve renal injury	Chang et al. (2018)
Triptonide	Wnt/*β*-catenin signaling	Relieve renal injury	Chinison et al. (2016)
AS-IV	Wnt/*β*-catenin signaling	Ameliorates fibrosis	Wang et al. (2014a)
Quercetin	Wnt/*β*-catenin signaling	Ameliorates fibrosis	Ren et al. (2016)

## Concluding Remarks

In summary, Wnt/*β*-catenin signaling plays a role in the pathogenesis of renal interstitial fibrosis, and targeting this pathway to treat renal fibrosis could yield positive results. Accumulating evidence has demonstrated that the role of Wnt/*β*-catenin signaling in the process of kidney repair and regeneration after AKI cannot be ignored (Kuure et al., 2007; Lasagni et al., 2015; Zhou et al., 2016; Jiao et al., 2017), and activating Wnt/*β*-catenin signaling could alleviate AKI (Li et al., 2020). A moderate increase in Wnt/*β*-catenin signaling is beneficial, but excessive activation of this pathway could trigger renal fibrosis (Guo et al., 2019; Sun et al., 2020). In addition, early intervention, but not late with β-catenin inhibitor significantly attenuates the apoptosis and inflammation induced by aristolochic acid (AA) (Kuang et al., 2021). Therefore, the dual role of Wnt/*β*-catenin signaling in CKD needs further study. And more researches are required to determine whether this pathway should be augmented in AKI to CKD. If so, the optimal treatment duration and the safe and effective dose also need to be determined.

At present, there are many studies on the Wnt/*β*-catenin signaling pathway. However, the mechanism of the non-canonical Wnt pathway in renal fibrosis still needs further investigation; Additionally, there are few clinical studies targeting Wnt/β-catenin signaling pathway to treat renal fibrosis, which is also the direction of our future efforts. since an FZD receptor can interact with different Wnt ligands to activate the Wnt pathway, coupled with the presence of several Wnt ligands. Therefore, understanding the selective binding of each Wnt protein to a specific FZD receptor and the resulting cascade reaction requires further research; Many signaling pathways play an important role in the process of renal fibrosis. However, the studies on the mechanism of their synergy with the Wnt/*β*-catenin signaling pathway should be further explored. In addition, a better understanding of the interactions between these pathways is needed to identify key molecules that regulate their interactions, which may serve as potential therapeutic targets; More small molecules that inhibit Wnt/*β*-catenin signaling are in development, which will explore more strategies to regulate this signaling pathway and provide more options for the effective therapies of renal fibrosis. The traditional Chinese medicine compound formula for the effective treatment of renal fibrosis is complex, and the active ingredients of Chinese medicine monomers or extracts are unknown. Therefore, the advantages of emerging natural products, CHMs, or new drugs in anti-renal fibrosis still need long-term research. Furthermore, to fully clarify the therapeutic effect of natural products in renal fibrosis, there is an urgent need to conduct studies that pay attention to identifying active ingredients, exploring action mechanisms, and rigorous pharmacological evaluation to ensure safety and accord the standards for clinical use. In conclusion, Wnt/*β*-catenin provides a broad prospect for treating renal interstitial fibrosis, but the understanding of Wnt/*β*-catenin remains a significant challenge.

## Glossary

α-SMA: α-smooth muscle actin
AA: aristolochic acid
AKI: acute kidney injury
APC: adenomatous polyposis coli
API: apigenin
CB2: cannabinoid receptor type 2
CBP: CREB-binding protein
CHMs: Chinese herbal medicines
CK1: casein kinase 1
CKD: Chronic kidney disease
CRD: cysteine‐rich domains
CRF: chronic renal failure
DAMPs: danger-associated molecular patterns
DKK1: Dickkopf1
DN: diabetic nephropathy
DVL: Dishevelled
ECM: extracellular matrix
EMT: epithelial-mesenchymal transition
ESRD: end-stage renal disease
fsp1: fibroblast-specific protein 1
FGF-2: fibroblast growth factor-2
FSGS: glomerulosclerosis
FZD: Frizzled
GSK3β: glycogen synthase kinase 3β
HH: Hedgehog
HIF-1α: hypoxia inducible factor-1α
IDO: indoleamine-2,3-dioxygenase
IRI: ischemia-reperfusion injury
IWR: inhibitors of Wnt response
LEF: lymphatic enhancer-binding factor
LRP5/6: low-density lipoprotein receptor-related protein-5/protein-6
MMP-7: matrix metalloproteinase-7
PAA: Poricoic acid A
PAI-1: plasminogen activator inhibitor-1
PGE2: prostaglandin E2
PORCN: Porcupine
(P)RR: (pro)renin receptor
PZC: poricoic acid ZC
PZD: poricoic acid ZD
PZE: poricoic acid ZE
PZG: poricoic acid ZG
PZH: poricoic acid ZH
RAS: renin-angiotensin system
SCIs: Substrate competitive inhibitors
Sfrp1: secreted frizzled protein 1
Snail1: Snail family zinc finger 1
SSc: Systemic sclerosis
tPA: tissue-type plasminogen activator
TCF: transcription factors T cell factor
TCM: traditional Chinese medicine
TECs: tubular epithelial cells
TGF-β: transforming growth factor β
Tnks: Tankyrase
TRPC6: transient receptor potential canonical 6
UA: uric acid
Upa: urokinase-type plasminogen activator
UUO: unilateral ureteral obstruction
Wif-1: Wnt inhibitor 1
Wnt/Ca^2+^ pathway: Wnt/calcium pathway
Wnt/PCP pathway: Wnt/planar cell polarity pathway
